# A Comprehensive Review of Bioactive Tannins in Foods and Beverages: Functional Properties, Health Benefits, and Sensory Qualities

**DOI:** 10.3390/molecules30040800

**Published:** 2025-02-09

**Authors:** Fernanda Cosme, Alfredo Aires, Teresa Pinto, Ivo Oliveira, Alice Vilela, Berta Gonçalves

**Affiliations:** 1 Chemistry Research Centre-Vila Real (CQ-VR), University of Trás-of-Montes e Alto Douro, Quinta de Prados, 5000-801 Vila Real, Portugal; avimoura@utad.pt; 2 Centre for the Research and Technology of Agro-Environmental and Biological Sciences (CITAB), Institute for Innovation, Capacity Building and Sustainability of Agri-Food Production (Inov4Agro), University of Trás-of-Montes e Alto Douro, Quinta de Prados, 5000-801 Vila Real, Portugal; alfredoa@utad.pt (A.A.); tpinto@utad.pt (T.P.); ivo.vaz.oliveira@utad.pt (I.O.); bertag@utad.pt (B.G.)

**Keywords:** proanthocyanidins, condensed tannins, hydrolyzable tannins, bioactive tannins, bioavailability, astringency, bitterness, nutritional quality, beverage, food

## Abstract

Tannins, a diverse class of polyphenolic compounds, are widely present in a variety of plant-based foods and beverages, where they contribute significantly to flavor, astringency, and numerous health benefits. Known for their antioxidant, anti-inflammatory, and cardioprotective properties, tannins are associated with a reduced risk of chronic diseases such as cardiovascular disease, cancer, and diabetes. Their bioavailability and metabolism are influenced by factors such as polymerization, solubility, and interactions with the gut microbiota. Tannin-rich beverages, including tea, wine, fruit juices, and cider, offer a range of health-promoting effects, including antioxidant, cardioprotective, and antimicrobial activities. In addition, tannins contribute significantly to the sensory and nutritional characteristics of fruits, nuts, and vegetables, influencing flavor, color, and nutrient absorption. The levels and efficacy of tannins are subject to variation due to factors such as ripeness and food processing methods, which can increase their impact on food quality and health. This review provides a comprehensive examination of the bioactive roles of tannins, their nutritional implications, and their sensory effects, highlighting their importance in both dietary applications and overall well-being.

## 1. Introduction

Tannins, a class of polyphenolic compounds and major secondary metabolites in plants, are essential in defining the sensory characteristics and nutritional quality of beverages, fruits, vegetables, and other plant-based foods. These compounds are abundant in plant-derived foods, particularly in cereals, nuts, chocolate, legume seeds, fruits, and vegetables, as well as in beverages such as wine, cider, tea, and cocoa [1]. Tannins significantly influence the sensory attributes of beverages and foods, especially astringency, bitterness, aroma, and mouthfeel. Although they offer considerable health benefits, high tannin levels can intensify bitterness and astringency, potentially impacting consumption [2].

**Figure 1:**
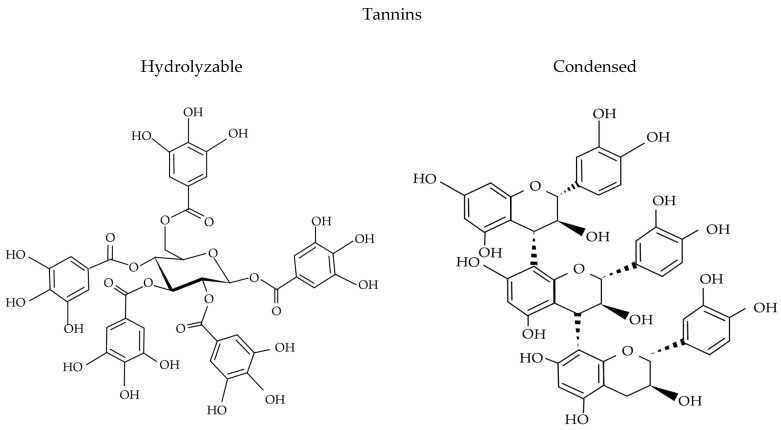
Main chemical structures of tannins.

Tannins are classified into hydrolyzable tannins, condensed tannins, complex tannins, and pseudo-tannins [3], as shown in Figure 1. Hydrolyzable tannins consist of a polyol core, often glucose, esterified with phenolic acids like gallic or ellagic acid. They are subdivided into gallotannins, such as tannic acid, and ellagitannins, which are formed through the oxidative coupling of gallic acid residues. These tannins are easily hydrolyzed under acidic, basic, or enzymatic conditions, releasing phenolic acids and exhibiting strong antioxidant activity due to their hydroxyl-rich structure [3,4].

In general, condensed tannins, also named proanthocyanins, are polymers of flavan-3-ol units (e.g., catechin, epicatechin) linked by carbon–carbon bonds, making them resistant to hydrolysis. Complex tannins are rare hybrids of hydrolyzable and condensed tannins, while pseudo-tannins are low-molecular-weight phenolic-like gallic acid and catechol that exhibit tannin-like reactivity but lack polymeric structures. All tannins share a polyphenolic backbone with abundant hydroxyl groups, which allows them to interact with proteins, polysaccharides, and metals. Their structural diversity supports applications in nutrition, medicine, and industry by providing antioxidant benefits and influencing bioavailability and biological activity, thereby broadening their practical applications [3,4,5,6,7,8].

More specifically, proanthocyanidins are composed of flavonoids such as flavan-3-ol or flavan-3,4-diol without a sugar core, whereas hydrolyzable tannins consist of esters of ellagic acids (ellagitannins) or gallic acids (gallotannins) bound to a sugar moiety, mainly glucose [3,4]. Hydrolyzable tannins have polyphenolic cores with molecular weights ranging from 500 to 3000 daltons (Da). In contrast, proanthocyanidins are formed by oligomeric or polymeric flavan-3-ol units, specifically (+)-catechin/(−)-epicatechin, (+)-gallocatechin/(−)-epigallocatechin, and (+)-afzelechin/(−)-epiafzelechin. These monomers differ based on the hydroxylation pattern of their A and B rings and in their C3 stereochemistry. The degree of polymerization determines the size of the proanthocyanidin, which can also be esterified with gallic acid to form 3-*O*-gallates. The structure of proanthocyanidins depends on various factors, including the type of flavan-3-ol unit, the linkage type, the degree of polymerization, spatial configuration, and whether the hydroxyl group is substituted [5].

Flavan-3-ol monomers can polymerize to form proanthocyanidins, which are classified as oligomers (dimers, trimers, and tetramers) or polymers with more than five subunits [6]. Oligomers and polymers composed exclusively of (+)-catechin/(−)-epicatechin are termed procyanidins, while those containing (+)-afzelechin/(−)-epiafzelechin or (+)-gallocatechin/(−)-epigallocatechin subunits are termed propelargonidin and prodelphinidin, respectively [7,8]. Procyanidins are the most abundant in plants, while propelargonidins and prodelphinidins are less abundant. These compounds have molecular weights ranging from 1000 to 20,000 Da [3,9] and are characterized by their aromatic rings and conjugated double bonds [10].

**Figure 2:**
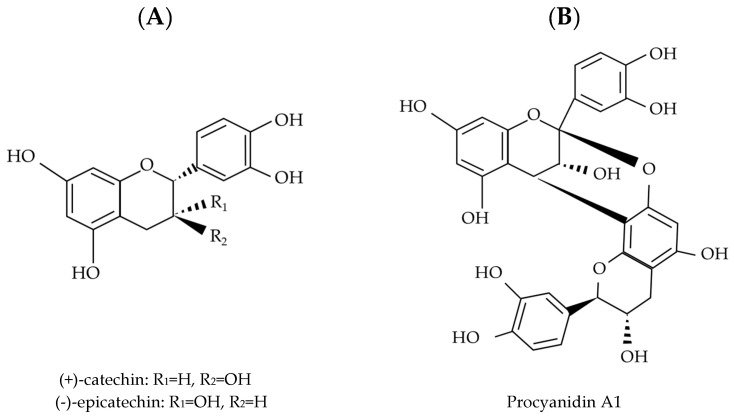

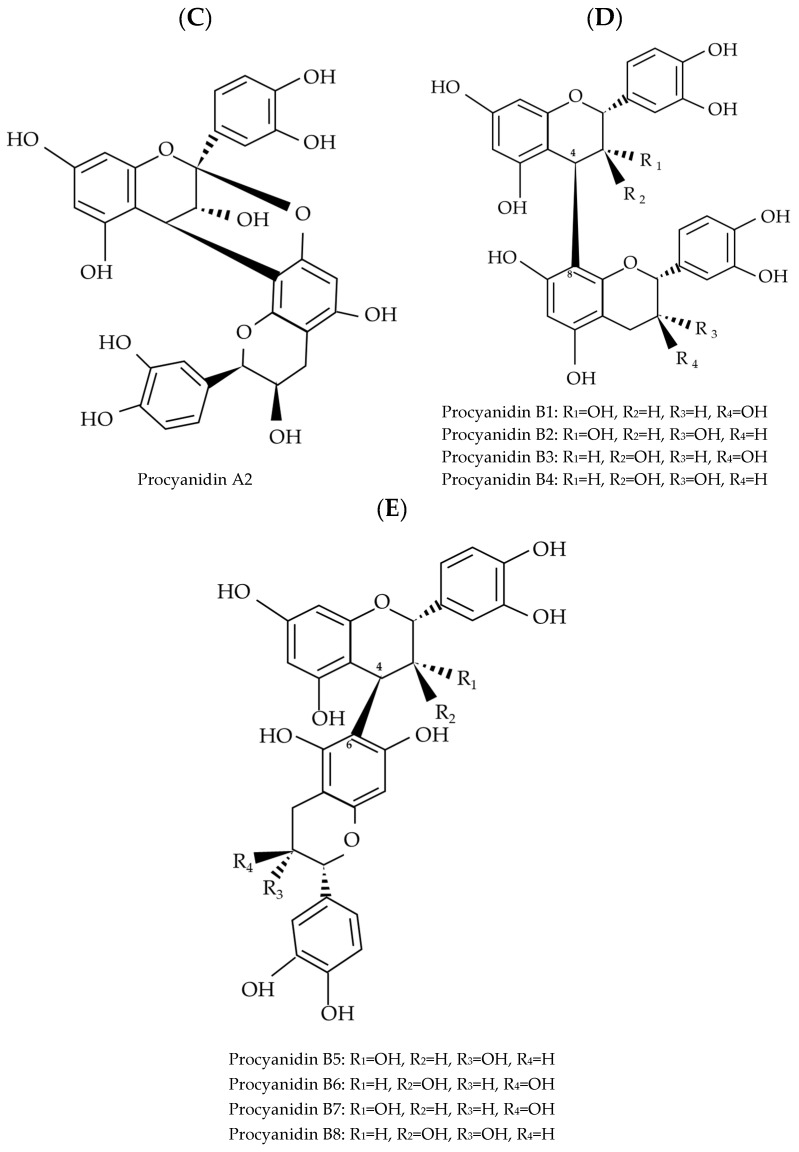
Structure of procyanidins. (**A**) Monomers, (**B**,**C**) A-type-dimer procyanidins, (**D**) B-type-dimer procyanidins linked with C4 → C8, and (**E**) B-type-dimer procyanidins linked with C4 → C6.

B-type proanthocyanidins are formed through oxidative condensation between the C4 carbon of the heterocyclic ring and the C6 or C8 carbons of adjacent units. The most common B-type dimers, B1, B2, B3, and B4, are associated with C4-C8 chemical bonds, while B5, B6, B7, and B8 contain C4–C6 interflavan bonds [4], as shown in Figure 2. In contrast, A-type proanthocyanidins have double bonds involving the C2 and C4 carbons of the upper unit and oxygen bonds at the C7 and C6 or C8 positions [7]. Nearly half of all plant-derived foods serve as dietary sources of proanthocyanidins. The composition of these compounds varies based on the constituent flavan-3-ol units, degree of polymerization, and type of interflavan linkage, all of which can affect their bioavailability, metabolism, and physiological effects [7].

The growing interest of consumers and food manufacturers in tannins is driven by their biological activities [11]. Epidemiological studies suggest that the consumption of tannin-rich foods and beverages may reduce the risk of certain diseases. Evidence suggests that tannins contribute to human health through anti-inflammatory, immune-regulatory, hypoglycemic, and hypolipidemic effects, as well as metabolic regulation, DNA repair, and anticancer properties [12]. Tannins provide health benefits either as complex, nonabsorbable structures with localized effects in the gastrointestinal tract (e.g., antioxidant, antimutagenic, free radical scavenging, antiviral, antimicrobial, and antinutrient activities) or as absorbable low-molecular-weight compounds and metabolites from colonic fermentation, which can have systemic effects on various organs [8].

Including tannins in the diet may help prevent cardiovascular disease, cancer, and diabetes while supporting gut microbiota and cognitive function [13,14,15]. They are particularly beneficial for cardiovascular health by inhibiting the oxidation of low-density lipoproteins (LDLs), commonly referred to as “bad” cholesterol, thereby reducing the risk of atherosclerosis. Furthermore, tannins may enhance cardiovascular function by lowering blood pressure, reducing LDL cholesterol levels, and improving vascular health [16]. However, the excessive intake of tannins may inhibit iron absorption, potentially posing a risk to individuals with iron deficiency [17].

Tannins are known for their antioxidant properties and are widely used in food applications. Numerous studies have extensively investigated their antioxidant activity, suggesting that they may help prevent cardiovascular disease, cancer, and osteoporosis [18].

This review aims to comprehensively explore the bioactive properties of tannins in foods and beverages, highlighting their functional roles and health benefits. It also examines their presence and effects in various beverages, fruits, nuts, and vegetables, focusing on their contributions to flavor, astringency, and overall sensory quality.

## 2. Functional Properties and Health Benefits of Dietary Tannins

Tannins are characterized by their ability to interact with macromolecules, influencing their functional roles in both food and biological systems. Due to their high polyphenolic content, tannins are considered potent antioxidants, effectively scavenging free radicals, reducing oxidative stress, and inhibiting lipid peroxidation [19]. This antioxidant property extends to their interaction with dietary proteins, affecting nutrient digestibility and bioavailability [20]. Additionally, tannins can chelate metal ions such as iron and copper, which catalyze oxidative reactions [21]. By reducing the availability of these metals, tannins help to mitigate oxidative damage in biological systems.

Tannins are also known for their antimicrobial activity, which includes disrupting microbial cell walls, inhibiting enzymes, and interfering with microbial adhesion [22]. This property is particularly valuable in food preservation and the treatment of infectious diseases. Moreover, tannins can inhibit digestive enzymes such as amylase, protease, and lipase [23], making them potentially useful in therapeutic applications like glycemic control in diabetes. Tannins influence weight management by modulating lipid metabolism and appetite, inhibiting pancreatic lipase, and reducing fat absorption. Their interactions with gut hormones may regulate hunger and satiety [24]. Proanthocyanidins in grape seeds have been shown to reduce amyloid-β aggregation, a key feature of Alzheimer’s disease.

In addition to these properties, recent studies have shown that tannins may modulate nitric oxide levels, thereby improving vascular health [25]. Foods rich in tannins, such as tea, wine, and certain fruits, may improve human health by lowering blood pressure and arterial stiffness, thereby reducing the risk of cardiovascular disease. Ellagitannins, an important group of tannins found in pomegranates and berries, have been associated with the prevention of tumor and carcinogenic processes [26].

Tannins also interact with the gut microbiota, modulating its composition and activity to promote the growth of beneficial bacteria such as *Lactobacillus* and *Bifidobacterium* [27] while inhibiting pathogenic microbes. Additionally, tannins improve gut barrier integrity and reduce inflammation, contributing to overall gastrointestinal health [28].

## 3. The Bioavailability and Metabolism of Tannins in the Human Body

Research on the bioavailability and metabolism of tannins remains limited, but these properties are closely related to their chemical and biological degradation, polymerization, and solubility [29,30]. Highly polymerized proanthocyanidins, in contrast to catechins, exhibit poor absorption through the gut barrier and minimal metabolism by the intestinal microflora. Compounds detected in blood or urine often differ significantly from those originally ingested. No specific intestinal receptors for tannin transport have been identified, suggesting that tannins and their precursors may rely on passive diffusion for absorption. However, tannin absorption in the stomach is minimal. In vitro studies suggest that procyanidins are hydrolyzed to more bioavailable monomers, although further in vivo confirmation is needed. Despite an estimated absorption rate as low as 10%, tannins may exert significant biological effects [31,32,33,34].

Tannins are often classified as antinutritional agents due to their ability to bind proteins and enzymes or interfere with iron and mineral absorption. Nevertheless, tannin-rich foods like wine offer health benefits. For instance, the beneficial effects of resveratrol in wine are partly attributed to tannins like procyanidins. Tannins exert their biological effects in two ways: (1) as unabsorbed complex structures acting in the gastrointestinal tract with antioxidant, antimicrobial, and antinutritional properties or (2) as low-molecular-weight tannins and microbial metabolites exerting systemic effects in various organs [33,34,35,36]. The bioconversion of ellagitannins by gut microbiota into bioavailable urolithins is associated with significant health benefits, including anti-inflammatory, anticancer, antiglycative, antioxidant, and antimicrobial effects observed in vitro. In contrast to hydrolysis by gastric acid, most tannins are metabolized by colonic bacteria [33]. Urolithins can be detected in urine approximately one week after ellagitannin consumption, highlighting the critical role of the gut microbiota in their bioavailability. Variations in microbiota composition directly influence urolithin production [37,38]. Ellagitannins have low bioavailability due to their large size and polarity. Furthermore, some tannins bind to specific proteins in the oral cavity and stop their metabolism [39]. These compounds are resistant to acid or basic hydrolysis in the gut, releasing only small amounts of ellagic acid. Limited absorption occurs in the stomach and small intestine, resulting in low concentrations of ellagitannin derivatives in plasma and urine [39,40].

Studies [41] using human Caco-2 cell lines as an intestinal absorption model indicate low permeability (Papp) values for hydrolyzable tannins and their hydrolysates, suggesting that absorption occurs primarily by passive diffusion, although proteins like organic anion-transporting polypeptide (OATP) and sodium/glucose cotransporter 1 (SGLT1) may play a role. Gastric digestion results vary between in vitro and in vivo studies; for example, while proanthocyanidins from chocolate hydrolyze to flavanol monomers under simulated gastric conditions, in vivo tests show limited depolymerization, probably due to buffering effects that reduce acid exposure [42]. During digestion in the small intestine, high-molecular-weight proanthocyanidins may form complexes with proteins, starches, or enzymes, reducing digestibility. Notably, one mole of proanthocyanidins can bind to twelve moles of protein, and smaller tannins with less complexation are absorbed more efficiently. Permeability coefficients for catechin and small proanthocyanidin dimers and trimers are comparable to mannitol, a paracellular transport marker, whereas oligomers with higher polymerization (e.g., a degree of six and a molecular weight of 1740) have much lower permeabilities. Nonetheless, indirect evidence suggests that proanthocyanidins are absorbed in the small intestine, as their in vitro effects are often mirrored in vivo [43,44,45,46,47,48].

Hydrolyzable tannins are degraded to gallic acid, pyrogallol, phloroglucinol, acetate, and finally butyrate through the sequential action of several bacterial enzymes [30]. Colonic bacteria, such as lactobacilli with tannase activity, have been identified as key players in this process [49]. The degradation of ellagitannins has been proposed to occur via the hydrolysis of ellagic acid, followed by microbial conversion to urolithin. Significant variability in metabolite production and excretion patterns has been observed, related to differences in the colonic microbiota [50].

As mentioned above, most molecules found in the bloodstream or excreted differ from those initially ingested, highlighting the importance of identifying biomarkers of tannin consumption. Several biomarkers have been identified, including ferulic acid derivates for the metabolism of caffeic acid derivatives, metabolites of hydroxyphenyl acetic acid derivates, hydroxyphenyl propionic acid derivates, and vanillic, homovanillic, and hydroxyhippuric acids for polyphenols, including flavonoids and hydroxycinnamic acids, and urolithin B is recognized as a biomarker for ellagitannins [51].

Although recent research has advanced our understanding of tannins, the current data primarily suggest an association between tannin consumption and health benefits [36,52,53]. Further studies are necessary to clarify the effects of tannins, particularly as they are increasingly incorporated into phytocomplexes used by the general population, often without a full understanding of their potential efficacy or toxicity [54]. This need is underscored by the complex nature of tannin bioavailability and metabolism, which remains an issue requiring further investigation [55].

## 4. Tannins: Human Health Implications and Gut Health vs. Antinutritional Aspects

In recent decades, there has been considerable consumer and food industry interest in polyphenolic compounds, particularly tannins, and their effects on human health, with a focus on the intestinal microbiota [56]. Tannins are a regular part of our daily diet, with an estimated intake of 0.1–0.5 g per day [8], primarily through beverages such as wine, tea, cider, infusions, and cocoa, as well as dried fruits, vegetables, and other plant-based foods [56]. As high-molecular-weight compounds derived from the secondary metabolism of plants, tannins play protective roles against various biotic and abiotic stressors [36]. Research has extensively investigated their preventive properties against chronic human diseases [57] and their effects on gut health [56].

Tannins have been associated with cardioprotective properties, such as inhibiting the oxidation of low-density lipoproteins, which helps to reduce the risk of atherosclerosis [58]. As antioxidants, they neutralize free radicals and protect cells from oxidative stress and DNA damage. Tannins also possess anticancer properties, aiding in cancer prevention and serving as adjuvants in cancer therapy [33,59,60]. Additionally, they exhibit antimicrobial activity, showing efficacy against bacteria like *S. aureus*, *E. coli*, *Streptococcus pyogenes*, *Enterococcus faecalis*, *P. aeruginosa*, *Yersinia enterocolitica*, and *Listeria innocua* [61]. Furthermore, studies suggest that tannins have antiviral properties against pathogens like influenza A, HIV, herpes, norovirus, and papillomavirus [62]. Research indicates that tannins may promote the growth of beneficial gut bacteria or activate their metabolic functions, highlighting their potential as prebiotic substrates for gut microbes [63,64].

Given these benefits, many researchers have advocated the use of tannins as nutraceuticals, supported by studies on the role of the gut microbiota in the metabolism of these polyphenols [56]. However, evidence on their bioavailability and pharmacokinetics remains limited and sometimes controversial. For tannins to exert health benefits, they must first be bioavailable. Intestinal absorption is thought to be inversely correlated with their degree of polymerization, as intestinal microbiota metabolize them into monomers [53]. High-molecular-weight tannins are not absorbed intact from the intestine, and about 90% of the procyanidins from apple juice are recovered in ileostomy effluent, allowing them to enter the colon [65]. Studies have shown that intestinal microflora catabolize proanthocyanidins to phenyl valerolactone, phenylacetic, and phenylpropionic acids in the colon [53]. Thus, tannins are only partially bioavailable in the intestinal tract, with hydrolyzable tannins and proanthocyanidins being absorbed in the small intestine and additional absorption occurring in the colon [66]. More research is needed to fully understand the bioavailability of tannins and their health effects [36].

On the other hand, tannins are considered antinutrients due to their interference with digestion and nutrient absorption [67]. While proanthocyanidins are widely used as dietary supplements [68], more evidence is needed to confirm their safety and to assess potential long-term toxicity.

Under certain pH and temperature conditions, tannins can form complexes with essential nutrients, such as proteins, carbohydrates, and certain minerals, inhibiting their absorption. Several mechanisms have been proposed in animal toxicity studies: (i) the inhibition of substrates and microbial enzymes that limit microbial growth through tannin–protein interactions; (ii) effects on cell membranes, such as the inhibition of oxidative phosphorylation and the electron transport chain; and (iii) the chelation of essential ions such as iron and zinc [69]. Studies of legumes show that tannin–protein complexes reduce protein digestibility, decrease amino acid availability, and increase fecal nitrogen in animals [70]. However, excessive consumption of tannins from legumes has not been associated with toxicity in humans [67].

Studies of pomegranate juice absorption showed low plasma concentrations of ellagic acid three hours after ingestion, and no intact ellagitannins were detected [71]. Ellagic acid depletion was found in plasma and urine, attributed to its poor water solubility and tendency to form complexes with magnesium and calcium ions, which inhibit absorption [8].

Tannins exhibit a dual function, acting locally in the gastrointestinal tract and systemically through their metabolites, which has stimulated interest in their potential therapeutic applications in inflammatory, metabolic, and oxidative conditions. Ongoing research is essential to better understand the interactions of these compounds in the body and their overall impact.

Recent evidence highlighting the health benefits of tannins suggests that their classification as antinutrients should be reconsidered. Comprehensive data from randomized controlled trials, long-term observational studies, and post-marketing analyses are essential to accurately assess their risks and benefits.

## 5. Tannins in Beverages, Fruit Juices, Fresh Fruits, Nuts, and Vegetables

### 5.1. Tea

Tannins significantly influence the flavor, quality, and health benefits of tea. Derived from the *Camellia sinensis* plant, tea is one of the most popular and widely consumed beverages worldwide. It is known for its distinctive flavor, health-promoting properties, and unique ability to both stimulate and relax. Tea also has deep cultural significance [72].

Green, black, and oolong teas are all made from the leaves of *Camellia sinensis*, with different processing methods resulting in different types of tea. Fresh tea leaves are rich in polyphenols, specifically flavan-3-ols, including (−)-epicatechin, (−)-epigallocatechin, (−)-epigallocatechin gallate, and (−)-epicatechin-3-gallate. Green tea made from unfermented, dried leaves is predominantly composed of polyphenols, which can account for up to 35% of its dry weight, with 60–80% being flavan-3-ols. In contrast, oolong and black teas, which are derived from partially or fully fermented leaves, contain about half the flavan-3-ol content of green tea [73].

Epigallocatechin-3-gallate is the most abundant flavan-3-ol in green tea, accounting for 50–80% of the total flavan-3-ol content, and is considered the most bioactive compound [74]. During the fermentation of black tea, monomeric polyphenols are converted into polymeric forms known as theaflavins and thearubigins. These polymeric compounds are less readily absorbed in the gastrointestinal tract compared to smaller catechins like (−)-epigallocatechin gallate. Consequently, black tea contains lower levels of monomeric polyphenols (3–10% of solids) and higher concentrations of polymers (23–25% of solids) than green tea. Oolong tea, which is partially oxidized, has intermediate levels of polymeric polyphenols and higher concentrations of (−)-epigallocatechin gallate [75].

Tea tannins are powerful antioxidants that neutralize free radicals and protect cells from oxidative damage [76]. These antioxidant properties have been linked with a reduced risk of chronic diseases, including heart disease and cancer [77,78]. Key bioactive compounds in tea, particularly flavan-3-ols (catechins) and tannins (theaflavins), exhibit potent free radical scavenging properties, helping to reduce oxidative stress and potentially prevent conditions like cardiovascular disease, cancer, and neurological disorders [79]. In addition, these compounds offer various health benefits, including anti-allergic, antibacterial, anti-cholesterol, antidiabetic, and anti-fungal effects [80]. These benefits are largely attributed to their antioxidant and metal-chelating properties [81].

Tea is appreciated not only for its taste but also for its extensive health benefits. Tannins contribute significantly to its astringency, bitterness, color, and antioxidant properties [82]. The astringency of tannins may also support digestive health by reducing diarrhea and acting as an effective protein precipitant in the intestine [83,84].

### 5.2. Wine

Wine is one of the oldest and most widely consumed alcoholic beverages in the world. Numerous studies have shown that moderate wine consumption is beneficial to health, protecting against neurological diseases, diabetes, cancer, and cardiovascular diseases [85], as shown in Table 1. These health benefits have been particularly highlighted in findings related to the “French paradox” [86]. Moderate red wine consumption has been associated with several health benefits, largely attributed to its phenolic compounds, particularly procyanidins, which include oligomeric and polymeric flavan-3-ols [87]. Several studies have shown an association between moderate daily wine consumption and a reduced risk of coronary heart disease. Khan et al. [88] investigated the effects of oligomeric procyanidins on vascular endothelial function, providing insight into the lower incidence of coronary heart disease observed in red wine consumers. Their research showed that oligomeric procyanidins induce atheroprotective changes in vascular function through oxidant signaling mechanisms originating from the mitochondrial electron transport chain. Another study investigated the relationship between moderate red wine consumption and glucose levels and diabetes. This study included 205 participants, with 101 red wine drinkers and 104 abstainers, with an average age of 60 years. The results showed that red wine drinkers had significantly lower rates of diabetes and lower glucose levels compared to abstainers [89]. Additionally, Vilahur and Badimon [90] reviewed the cardioprotective effects of the Mediterranean diet on coronary heart disease and atherosclerosis. They concluded that moderate daily consumption of red wine, for example—0.15 L for women—reduced inflammation and atherosclerosis while improving lipid metabolism, antioxidant capacity, and endothelial function. Red wine has also been shown to have antibacterial activity against *Helicobacter pylori*. These beneficial effects are primarily attributed to the flavonoids found in red wine [91].

The health benefits of polyphenols in wine are influenced by both the amount consumed and their bioavailability. According to Basalekou et al. [104], the total tannin content in wine ranges from 1.1 to 3.4 g/L, while Chira et al. [105] reported a slightly narrower range of 1.2 to 2.2 g/L. The composition of proanthocyanidins in wine is influenced by climatic and geographical conditions, viticultural practices, and the stage of ripeness of the grape. Flavan-3-ols in wine are present in both monomeric forms and in oligomeric and polymeric forms. The concentration of the monomeric form in wine typically ranges from 40 to 120 mg/L [106,107].

In red wine, the concentration of oligomeric and polymeric forms, also known as condensed tannins or proanthocyanidins, varies from 500 to 1500 mg/L, depending on the age of the wine. In white wine, on the other hand, these compounds are present in concentrations ranging from 10 to 50 mg/L [106,107].

Furthermore, phenolic content and composition can vary significantly depending on the grape variety, with notable differences in monomers ((+)-catechin/(−)-epicatechin and (+)-gallocatechin/(−)-epigallocatechin), oligomers (dimers, trimers, and tetramers), or polymers (Table 2). Hydrolyzable tannins are not naturally present in *Vitis vinifera* grapes but are introduced during the fermentation and aging process in barrels, which are particularly rich in these tannins, especially ellagitannins [108]. The final concentration of these tannins in wine can range from 0.4 to 50 mg/L [109]. Wines aged in wooden barrels may have increased levels of hydrolyzable tannins, especially ellagitannins, as shown in Table 2.

As a result, the total phenolic content in a typical glass of red wine is approximately 100–200 mg, compared to 25–50 mg in a glass of white wine [106].

The wide range of phytochemicals in red wine makes it difficult to identify the specific compounds responsible for its protective effects. However, research has linked oligomeric proanthocyanidins to improved vascular health. Procyanidins have been identified as the primary vasoactive polyphenols in red wine [117] and play a key role in providing cardiovascular benefits. High polyphenol intake has been shown to inhibit atherosclerosis in experimental models [118]. Red wine polyphenols promote the endothelium-dependent vasodilation of blood vessels and suppress the synthesis of endothelin-1 (ET-1), a vasoconstrictor peptide [119], which may contribute to their anti-atherosclerotic effects. In addition, B-type oligomeric procyanidins in wine have been correlated with reduced ET-1 synthesis [117]. However, a consensus on the protective effects of red wine remains elusive, possibly due to variations in vasoactive compounds between different wines.

Red wine also plays a role in preventing fat oxidation during digestion, offering a novel strategy to reduce cardiovascular risks associated with oxidized fats, such as those from beef [120]. Wine procyanidins are particularly effective in preventing lipid oxidation in the digestive tract, thereby reducing postprandial increases in plasma oxidants. Despite the limited bioavailability of these compounds, they are essential for optimizing nutrition and reducing the risk of atherogenesis [121]. The gastrointestinal tract is considered the primary site of action for dietary antioxidants, which act as chemical antioxidants. Therefore, wine polyphenols may inhibit the production and absorption of toxic lipid oxidation products, thereby counteracting postprandial oxidative and associated inflammatory responses [122].

Furthermore, a recent study [123] investigated the effects of a mixture of ellagitannins derived from oak bark (*Quercus petraea* L.) on cardiovascular, metabolic, and liver health in both spontaneously hypertensive rats and rats on a high-fat diet. The study found that tannin extracts improved cardiovascular, metabolic, and hepatic function, suggesting that ellagitannins from oak bark may enhance the beneficial effects of red wine.

### 5.3. Fruit Juices

The concentration of proanthocyanidins in various fruit juices has been documented in several studies. For instance, apple juice contains proanthocyanidin concentrations ranging from undetectable levels to 690 mg/L [36,124]. Pear juice has concentrations ranging from 1450 to 4060 mg/L depending on the variety and the degree of maturity [125], while grape juice concentrations range from 3.5 to 470 mg/L [36]. Cranberry has been reported to contain proanthocyanidin concentrations between 200 and 210 mg/L [36].

Evidence suggests that the consumption of fruit and vegetable juices may be as effective as the consumption of whole fruits and vegetables in reducing the risk of chronic disease [126]. Furthermore, individuals with a higher intake of fruit and vegetable juices have been shown to have a significantly lower incidence of Alzheimer’s disease [127]. Juices rich in ellagitannins include those from pomegranates, strawberries, raspberries, and blackberries [128]. Pomegranate juice, in particular, has received considerable attention for its health benefits [129]. It is rich in tannins and has anti-atherosclerotic, anti-aging, antioxidative, and anti-hypertensive properties [130]. Studies have shown that pomegranate juice contains higher levels of antioxidants than other natural juices, with antioxidant activity three times higher than that of red wine or green tea [131]. These antioxidants offer several benefits, including protection against cholesterol oxidation, anti-aging effects, and the prevention of atherosclerosis [132].

In human studies, pomegranate juice consumption has been shown to reduce low-density lipoprotein (LDL) aggregation and increase serum paraoxonase activity by up to 20% [133]. Paraoxonase, an esterase associated with high-density lipoproteins (HDLs), plays a critical role in protecting lipids from peroxidation [134]. Clinical trials in patients with hypertension and/or obesity have shown that the consumption of pomegranate juice can reduce systolic and diastolic blood pressure [129,135] while increasing high-density lipoprotein (HDL) cholesterol levels.

Pomegranate ellagitannins have been specifically studied for their cardioprotective effects in animal models [136]. Pomegranate juice has been shown to reduce isoproterenol-induced cardiac necrosis [137] and to reduce angiotensin-induced glucosuria, hypertension, and proteinuria in streptozotocin-induced diabetic rats [138].

Stone fruit juices from tart cherry, blackthorn, and cornelian cherry have also been analyzed for proanthocyanidin content. Among them, tart cherry juice showed the highest concentration at 1254.99 mg/L, while blackthorn and cornelian cherry juices contained 306.9 mg/L and 21.56 mg/L, respectively [139].

Cranberry juice (*Vaccinium macrocarpon*) is known for its ability to protect the urinary tract from pathogenic bacteria. Due to its high concentration of proanthocyanidins, it is frequently recommended for the treatment of urinary tract infections and prostatitis [140]. The bioactive compounds in cranberry juice are A-type proanthocyanidins, which inhibit the adhesion of *Escherichia coli* to the urinary tract epithelium, directly preventing bacterial biofilm formation [141].

Table 3 provides an overview of the monomers, dimers, oligomers, and polymers of flavanols found in various fruit juices.

### 5.4. Other Beverages

Native populations that consume significant amounts of cocoa on a daily basis have been observed to have lower incidences of hypertension, cardiovascular disease, obesity, diabetes mellitus, heart attack, stroke, and cancer [143]. These health benefits are attributed to the presence of oligomeric procyanidins, including dimeric procyanidins B1 to B7, trimeric procyanidin C1, tetrameric procyanidin (cinnamtannin A2), and pentameric procyanidin (cinnamtannin A3) [144]. These compounds are more effective than monomeric and polymeric cocoa procyanidins in providing health benefits [145].

Cider is traditionally recognized as a beverage made from fermented apple juice, with an alcohol content ranging from 1.2% to 8.5% [146]. Apples are rich in polyphenols, including flavan-3-ols such as catechins and procyanidins [147]. In terms of proanthocyanidins, apple cider consists mainly of procyanidins, which are composed of two diastereoisomeric monomeric configurations of flavan-3-ols: (+)-catechin and (−)-epicatechin [148]. The procyanidin content of cider is generally lower than that of fresh apples [148]. Cider contains two procyanidin monomers, four dimers, three trimers, and one tetramer [149].

Health benefits associated with apple polyphenols include reduced cancer risk, high antioxidant power, anti-inflammatory and anti-tumor properties, and the inhibition of carcinogenesis in tissues such as the skin, breast, and colon [150]. Cider is also recognized for its therapeutic properties, including antioxidant and antimicrobial activities, as well as its potential to increase longevity and reduce cardiovascular disease and type 2 diabetes [151]. The phenolic composition of cider is complex and varies significantly depending on factors such as the apple cultivar, ripeness, and fermentation strain, with the apple cultivar playing a critical role [150,152,153,154].

In beer, the primary monomeric flavanol detected is (+)-catechin (0.5 to 6.9 mg/L), with smaller amounts of (−)-epicatechin (0.8–1.9 mg/L), (−)-catechin gallate, (−)-epicatechin gallate, and two glycosides also identified [155,156]. Studies using thiolysis coupled with RP-HPLC-ESI (−)-MS/MS in beer polyphenolic oligomers [157] indicate that most beer dimers are procyanidin B3 (consisting of two catechin units), whereas most trimers are prodelphinidins (containing catechin terminal units and either gallocatechins or catechins as extension units). Four types of dimers have been identified: three procyanidins (B1, B3, and B4) and one prodelphinidin (B3) [158].

### 5.5. Fresh Fruits

Tannins are naturally occurring polyphenolic compounds in various fresh fruits (Table 4) that contribute to their taste, color, and potential health benefits [56]. These compounds are particularly abundant in grapes, apples, pears, berries, and stone fruits like peaches and plums [159,160,161]. For instance, a study conducted in the Ida Mountains, Turkey, found that sour cherries (*Prunus cerasus*) had the highest content of condensed tannins among various fruits [162], while greengage plums (*Prunus domestica*) of the “Papaz” variety had the lowest [162]. Pomegranates and persimmons are also rich sources of tannins, with unripe persimmons containing particularly high concentrations [163].

Tannins play an important role in plant defense mechanisms, protecting against predators and fungi [164,165,166,167]. In humans, tannins contribute to the astringent taste and dry mouth often associated with certain fruits and their derivatives, such as wine [97].

The tannin content of fruit can vary significantly depending on factors such as ripeness, growing conditions, and environmental stressors [168,169]. For instance, in Syrah grapes, an increase in skin tannins and a decrease in seed tannins have been observed with increasing altitude [169]. Moreover, in persimmons, soluble tannins are gradually converted to insoluble tannins as the fruit ripens, thereby reducing astringency [170]. This conversion occurs naturally in non-astringent persimmon cultivars, while astringent cultivars retain highly soluble tannins until they reach full ripeness [170]. In a ten-year field trial in Monnington-on-Wye, Herefordshire, significant variations in tannin content were found in apples from the same trees in different years. The cv. Tremletts had an average tannin content of 0.43%, while cv. Dabinett had an average of 0.27%. On the other hand, fruit grown under low nitrogen conditions or adverse weather tended to have higher tannin levels [166]. Research on cider apple trees showed that unshaded trees produced 32% more total polyphenols and 11% larger fruits than those shaded during early growth stages [171]. Strawberry cultivars grown in different European locations showed genotype and environment variations in tannin composition, particularly in the balance between proanthocyanidins and ellagitannins, influenced by specific environmental conditions [168].

Nutritionally, tannins in fruits act as antioxidants and may provide health benefits by reducing oxidative stress in the human body [172]. However, they can also inhibit the absorption of certain nutrients, particularly iron [34]. In addition, tannins often decrease protein digestibility and the availability of essential amino acids by forming reversible and irreversible tannin–protein complexes through interactions between the hydroxyl groups of tannins and the carbonyl groups of proteins [173].

In summary, tannins are an integral part of many fruits, influencing their sensory properties and potentially contributing to human health. Their presence and concentration in fruits reflect a complex interplay of genetic and environmental factors, making them an interesting subject for ongoing research in food science and nutrition.

**Table 4:** Tannin content in several types of fruits.

Fruit/Species	Tannins	Content	Reference
Tannic acid equivalents (TAEs)			
Apple	Total tannins	16.47 mg/g fw	[174]
Pomegranate	Condensed tannins	62.7–139.6 mg/g dw	[175]
Catechin equivalents (CEs)			
Apricot	Condensed tannins	138.8 ± 1.34 µg/g dw	[162]
Cherry	Condensed tannins	13.1 ± 5.8 µg/g dw	[162]
Grapes (Skins)	Condensed tannins	6–165 mg/g dw	[176]
Grapes (Seeds)	Condensed tannins	3–241 mg/g dw	[176]
Greengage Plum	Condensed tannins	20.42 ± 0.81 µg/g dw	[162]
*Prunus* sp.	Condensed tannins	2.2–37.6 mg/g dw	[177]
Sour Cherry	Condensed tannins	163.4 ± 3.21 µg/g	[162]
Gallic acid equivalents (GAEs)			
Persimmon cv. Cheongdo-Bansi	Hydrolyzable tannins	4.68 ± 0.09 g/kg dw	[170]
Persimmon cv. Daebong	Hydrolyzable tannins	4.97 ± 0.03 g/kg dw	[170]

fw—fresh weight; dw—dry weight.

### 5.6. Nuts

Tannins, a diverse group of polyphenolic compounds, are found in a wide variety of plant-based foods, including nuts. These complex biomolecules play an important role in plant defense mechanisms and human nutrition. In nuts, tannins contribute to flavor, color, and potential health benefits, while influencing nutrient absorption and digestibility [44]. The astringent sensation caused by tannins in nuts is due to their ability to bind and precipitate salivary proteins [178]. While this can affect palatability, it also contributes to the characteristic flavor profile of many nuts [179]. Tannins enhance the complex flavor profiles of nuts, especially when roasted. The Maillard reaction during roasting can alter tannin structures, creating new flavor compounds. Tannins can contribute to the color of nut products, especially in processed forms like nut butters or extracts [180]. Moreover, the antimicrobial properties of tannins can naturally preserve nuts, potentially extending their shelf life [181].

The tannin content of nuts varies considerably between species and even within cultivars of the same species (Table 5). For instance, acorns from oak trees (*Quercus* spp.) are known for their high tannin content. Studies of acorns from *Quercus robur* and *Quercus petraea* in Poland found tannin contents ranging from 2.4% to 5.2% of dry matter [181]. This high tannin content contributes to the astringency of raw acorns, necessitating processing (such as leaching) before consumption. Walnuts are unique for their hydrolyzable tannins and related compounds, such as ellagic acid and its derivatives [182]. Ellagitannins found in walnuts include pedunculagin, casuarinin, casuarictin, strictinin, tellimagrandin, praecoxin, rugosin, and isostrictinin [183]. These tannins, along with ellagic acid derivatives, are responsible for the astringent taste of walnut kernels [184]. In contrast, hazelnuts, pecans, and almonds contain both hydrolyzable and condensed tannins [183]. Studies on Turkish hazelnut samples identified several hydrolyzable tannins, including flavogallonic acid dilactone, valoneic acid dilactone, sanguisorbic acid dilactone, HHDP-glucose, and ellagic acid derivatives [185]. The concentration and types of proanthocyanidins may vary depending on how the nuts are processed, such as whether they are raw, roasted, or blanched [186]. Hazelnut skins are particularly rich in tannins, including catechin, epicatechin, and their oligomers. Hazelnuts also contain hydrolyzable tannins such as glansreginin A and glansreginin B [187]. Almonds, especially those with intact skins, contain significant amounts of tannins. Research shows that almonds with skins have a total phenolic content of 239 mg GAE/100 g fresh weight, compared to 47 mg GAE/100 g fresh weight for almonds without skins [188]. Pecans, pistachios, and cashews also contain tannins, though generally at lower levels than acorns or walnuts [183]. The mean and interquartile range (25% to 75%) of polyphenolic contents per 100 g of almonds reported in the literature [186] were 162 mg (67.1–257) of proanthocyanidins (dimers or larger), 82.1 mg (72.9–91.5) of hydrolyzable tannins, 61.2 mg (13.0–93.8) of non-isoflavone flavonoids, 5.5 mg (5.2–12) of phenolic acids and aldehydes, and 0.7 mg (0.5–0.9) of isoflavones, stilbenes, and lignans.

Tannin fractions from walnuts, hazelnuts, and almonds chelated copper and iron ions, whereas zinc was significantly bound only by almond and hazelnut tannins. Chelation efficiencies followed the order Cu(II) > Fe(II) > Zn(II), except for in almond tannins, which chelated more zinc than iron. Walnut tannins, which are rich in hydrolyzable tannins, showed low Zn(II) chelation but higher Cu(II) chelation compared to hazelnut and almond tannins, which mainly contained proanthocyanidins [189].

Beyond their role in food, tannins from nut skins and shells have potential applications in several industries—(i) leather tanning: historically, tannins from tree barks (including some nut trees) have been used in leather production [190]; (ii) natural dyes: tannins can be used as natural dyes in textile and other industries [191,192]; and (iii) pharmaceutical and cosmetic industries: the antioxidant properties of nut tannins are being investigated for potential use in these sectors [180].

Nut tannins are a complex and fascinating area of food science and nutrition. While they can present challenges regarding nutrient absorption, their potential health benefits as antioxidants and their contributions to flavor and food quality make them an essential component of nuts. As research continues, our understanding of the role of tannins in nuts and their impact on human health is likely to expand, potentially leading to new applications in the food, health, and industrial sectors.

**Table 5:** Tannin content in several nut species.

Nuts	Tannin	Content	Reference
Tannic acid equivalents (TAEs)			
Cashews and apple	Total tannins	193.29 ± 7.65 mg/100 mL	[193]
Pistachios	Total tannins	49.7–86.7 mg/g dw	[194]
Hazelnuts	Total tannins	40.4–357.8 mg/g dw	[195]
Catechin equivalents (CEs)			
Almonds	Condensed tannins	776 mg/g dw	[189]
Hazelnuts	Condensed tannins	1261 mg/g dw	[189]
Pistachios	Condensed tannins	211–307 mg/100 g	[195]
Walnut	Condensed tannins	147 mg/g	[189]
Gallic acid equivalents (GAEs)			
Pistachios	Hydrolyzable tannins	240 mg/100 g	[195]
Walnut (green fruit)	Hydrolyzable tannins	116.3 mg/g extract	[190]

fw—fresh weight; dw—dry weight.

### 5.7. Vegetables

Tannins are commonly found in vegetables, although their concentration varies widely from species to species. Although tannins were once considered antinutrients due to their ability to interfere with nutrient absorption, recent studies have highlighted their potential health benefits, including antioxidant, antimicrobial, and anticancer properties [67,196]. Vegetables contain varying amounts of tannins, with some having higher concentrations than others (Table 6). Common vegetables that contain tannins include beans, lentils, leafy greens, herbs, and spices, including mint, rosemary, coriander, and sage [197]. Red-colored beans tend to have the highest tannin content, while white-colored beans have the lowest. Chickpeas (garbanzo beans) also have a relatively low tannin content [198]. In a study [199] on fresh vegetables, *Amaranthus viridis* had the highest tannin content (30.20 ± 1.05 mg/100 g dry weight), while *Veronia amygdalina* had the lowest (1.02 ± 0.02 mg/100 g dry weight). In blanched vegetables, *Amaranthus viridis* still had the highest tannin content (46.81 mg/100 g dry weight), while *Corchorus oliterus* had the lowest (5.05 mg/100 g dry weight). However, in juiced extracts, *Launea taraxacifolia* had the highest tannin content (735.77 mg/100 g dry weight), while *Celosia argentea* had the lowest (67.18 mg/100 g dry weight). These results suggest that both juicing and blanching significantly affect the tannin content of vegetables. Other food processing techniques can affect the tannin content of vegetables. (i) Soaking can reduce the tannin content of legumes [67]. For instance, raw *Sphenostylis stenocarpa* contained 39.88 mg/g of tannins. Soaking the seeds at hydration levels of 10%, 25%, 50%, and 100% resulted in reductions in tannin content of 4.11%, 7.07%, 13.87%, and 14.29%, respectively [200]. (ii) Hydrothermal processing is another technique that can lower tannin levels in vegetables, potentially enhancing nutrient bioavailability. According to Ojo [67], all hydrothermal processing methods, including boiling and steaming at both atmospheric and elevated pressures, significantly affected the tannin content of legume seeds, with varying degrees of reduction. The lowest tannin content (6.85 mg/g) was observed in the white cultivar of *Mallotus subulatus*, which was boiled at normal atmospheric pressure. The highest value (8.15 mg/g) was obtained after steaming at elevated pressure. A similar reduction of 78.46% was observed for *Cassia hirsuta* when subjected to conventional and pressurized boiling (PB) [201].

In summary, tannins in vegetables are complex compounds that present both benefits and challenges [202]. Although they can partially interfere with nutrient absorption, they significantly enhance the antioxidant properties of vegetables [203]. Various processing methods can influence tannin levels, thereby impacting the nutritional profile of these foods. Ongoing research into the health implications of tannins highlights their vital role in a balanced, plant-rich diet, underscoring their importance as functional components of nutrition.

**Table 6:** Tannin content and composition in vegetables.

Vegetable	Tannin	Content	Reference
Beans	Condensed tannins	5–830 mg/100 g	[186]
Kidney beans	Total tannins	2.91–6.66 mg TAE/100 g	[202]
Chickpea	Total tannins	488.1 mg CE/100 gDM	[195]
Cowpea	Total tannins	390.9 mg CE/100 gDM	[195]
Faba bean	Total tannins	684.6 mg CE/100 gDM	[195]
Soybean	Total tannins	225.5 mg CE/100 gDM	[195]

TAEs: tannic acid equivalents; CEs: catechin equivalents.

## 6. Sensory Qualities Affected by Tannins

Tannins have a significant impact on the sensory qualities of wine and fruit, particularly on attributes such as astringency, bitterness, aroma, and overall mouthfeel.

While polyphenols are known for their health benefits, high tannins can contribute to bitterness and astringency, potentially impacting food consumption. Research suggests that taste often takes precedence over nutritional value in food choices [2]. This poses a challenge to the food industry in developing health-promoting foods. This has driven research efforts to identify bitter and astringent compounds, understand the impact of food matrices on tannin perception, and develop methods to modulate these tastes while preserving health benefits [204].

### 6.1. Astringency

**Figure 3:**
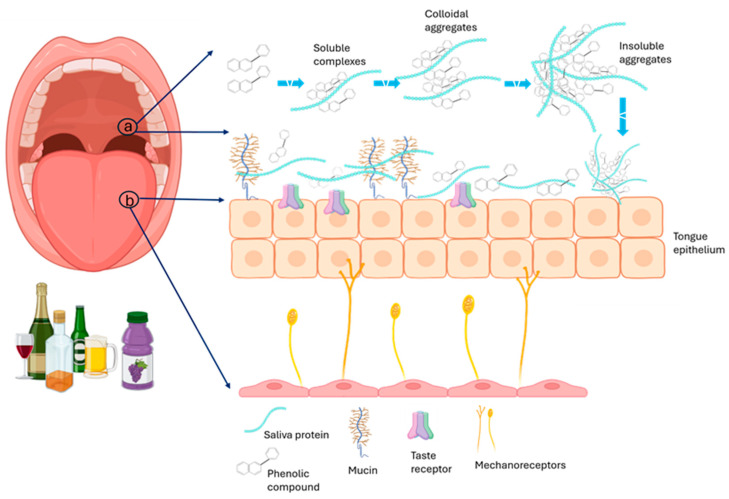
(a) Saliva protein and tannin interactions causing precipitation of salivary proteins; (b) interaction of tannins with oral cells and/or mucosal pellicle and activation of oral mechanoreceptors.

Astringency is a key sensory attribute of foods and beverages, particularly wine, characterized by sensations of dryness, roughness, and puckering. This complex mouthfeel is due in part to interactions between tannins and salivary proteins, resulting in the depletion of the lubricating salivary film and creating a dry rough sensation in the mouth [205,206]. Larger, more water-soluble tannins are perceived as more astringent, while smaller, more hydrophobic tannins tend to be perceived as hotter and more bitter [207]. Astringency is closely related to the interaction between tannins and salivary proteins, contributing to a reduction in the protective salivary film [205] (Figure 3).

A high tannin content and low pH influence specific sub-qualities of astringency, such as drying and puckering. The acidity of a substance plays a significant role in astringency, with high acidity (low pH) increasing the puckering sensation independently of the drying effect caused by tannins. These sub-qualities have different physicochemical origins [205].

The drying sensation is closely related to the boundary friction of saliva–wine (or saliva–unripe persimmon) mixtures. A higher tannin content increases the drying effect by interacting with the salivary protein film, resulting in higher surface-adsorbed mass without significant shear [205,208,209]. Puckering is primarily driven by low pH, which causes a faster collapse of the salivary film. This sensation is distinct from drying and is not directly influenced by tannin content [205,208,209]. Roughness is considered a secondary sub-quality induced by drying or puckering and does not scale with any specific physical measure explored in the cited studies [205,208].

In wine and other fruit-fermented beverages, overall astringency and its sub-qualities can vary significantly depending on the composition of the wine, including tannin levels, pH, and the presence of other components like polysaccharides such as mannoproteins [205,208,209]. Different astringent stimuli (e.g., alum, malic acid, tannic acid) can interact in complex ways. For instance, mixtures of tannic acid and malic acid show synergy for drying and roughing, whereas mixtures of alum and malic acid show antagonism for these sub-qualities [210].

A tribological approach studying friction, lubrication, and wear has been used to evaluate the mechanisms of astringency. Higher boundary friction correlates with the drying sensation, while the rate of friction increase is associated with puckering [208,209]. Modified progressive profiling techniques, such as Temporal Dominance of Sensations (TDS), have been used to capture the dynamic nature of astringency and its sub-qualities over time. This method helps in understanding how wines exhibit different astringency profiles at different test times [211]. In one study [212], the influence of protective colloids on calcium tartrate stability and astringency perception in red wine was determined. It was found that the presence of exogenous protective colloids did not affect the overall astringency rating of the wines but altered their dynamic perception according to their TDS profiles compared to untreated wine.

### 6.2. Bitterness

Bitterness is an important sensory attribute associated with tannins and polyphenolic compounds in various foods and beverages. Several polyphenols, including tannins, act as agonists for bitterness receptors [213]. The molecular structure of tannins influences bitterness, with more hydrophobic tannins contributing more to this sensation [207,213].

Tannins and other polyphenolic compounds activate specific human bitter taste receptors (TAS2Rs). Different tannins activate different combinations of these receptors, contributing to the bitterness perceived in foods and beverages like red wine, beer, tea, and chocolate [214].

In wines, bitterness is significantly correlated with the levels of tannins and other polyphenolic compounds [215]. The bitterness and astringency of wines are also influenced by factors such as pH, ethanol content, and the presence of other compounds [216]. The structure characteristics of tannins, such as polymer size and galloylation, influence their bitterness and astringency [217]. Additionally, the presence of dietary lipids can alter the sensory perception of tannins. For instance, consuming wine with foods high in lipids can reduce the perception of both astringency and potential bitterness [218].

### 6.3. Aroma, Flavor, and Overall Mouthfeel

Tannins can enhance or modify the aroma and flavor of wine. The addition of tannins, especially mixed tannins, can improve the sensory qualities and antioxidant activities of a wine [218]. Ellagitannins in particular have been shown to preserve the perception of fruitiness and prevent the development of oxidized notes in wines [219]. Grape tannins can also increase the levels of varietal thiols, which contribute to tropical aromas in wines like Sauvignon Blanc and enhance fruity and green notes [220]. During winemaking and aging, tannins undergo various chemical reactions that can lead to the formation of new aroma compounds or the stabilization of existing ones [221].

The addition of enological tannins can increase the complexity of wine aromas. For instance, grape seed tannins have been found to promote the formation of higher alcohols, thereby increasing aroma complexity in young red wines [222]. However, tannins can also reduce certain aroma compounds, such as acetate esters and volatile fatty acids, while increasing primary aromas derived from aroma precursors in grape skins [223].

As mentioned above, tannins have a significant effect on the flavor and mouthfeel of wine. Higher tannin concentrations increase the perception of bitterness, astringency, and dryness [224,225]. Tannins interact with other wine components like alcohol and mannoproteins, influencing the overall sensory perception of flavors and mouthfeel [226]. Tannins contribute to wine stability and protection against oxidation, which may indirectly influence flavor by maintaining the integrity of flavor compounds over time [219,221].

During fermentation, the use of non-*Saccharomyces* yeasts in combination with tannins can enhance wine color and anthocyanin composition, contributing to a more complex flavor profile [227].

Tannins influence mouthfeel attributes such as dryness, roughness, and gumminess. They are primarily responsible for the astringency of wines and contribute to the perception of dryness and roughness [213]. The interaction of tannins with salivary proteins can lead to different mouthfeel sensations [205].

## 7. Final Remarks

Tannins, a diverse class of polyphenolic compounds, offer remarkable sensory and health benefits. Their structural diversity significantly influences the flavor, astringency, and bioactivity of various foods and beverages. Known for their antioxidant, anti-inflammatory, and cardioprotective properties, tannins also have therapeutic applications, including metal chelation, the promotion of vascular health, and disease prevention. However, balanced consumption is essential to minimize potential problems with nutrient absorption while maximizing tannin benefits. Tannins, found in beverages like tea, wine, and fruit juices, contribute to health benefits such as antioxidant and cardiovascular protection. The different tannin profiles in these beverages reveal their potential to prevent chronic diseases, highlighting the importance of bioavailability and consumption patterns. Similarly, tannins in fruits, nuts, and vegetables offer both benefits and limitations. Despite their potential to inhibit nutrient absorption, their antimicrobial, anticancer, and antioxidant effects remain highly valuable. Although research on the bioavailability and metabolism of tannins has advanced, further studies are needed to fully understand their health implications. Beyond their physiological roles, tannins shape the sensory attributes of foods and beverages, particularly wine, affecting astringency, bitterness, aroma, and mouthfeel. Their complex interactions with other compounds present both challenges and opportunities for improving food quality while preserving their health-promoting properties.

## Figures and Tables

**Table 1:** Positive effects of moderate red wine consumption on health.

Diseases	Phenolic Compounds	References
Neurological diseases	Procyanidin	[92,93]
Diabetes	Monomeric, dimeric, trimeric, tetrameric, oligomeric procyanidins Proanthocyanidins	[89,94]
Cancer	Procyanidins B1, B2, B3, B4, B5	[95,96,97]
Cardiovascular diseases	Oligomeric procyanidins	[85,88,97,98,99,100]
Atherosclerosis	Proanthocyanidins	[90,101,102]
Antibacterial activity	Flavonoids	[91,103]

**Table 2:** Condensed and hydrolyzable tannins (mg/100 mL) in wine.

	Monomers	Oligomers	Polymers	Proanthocyanidins	Hydrolyzable Tannins	Ellagitannins	Reference
Wine	-			1–53		2–5	[36]
	Red						
Red wine	5.24	12.48		-	-	-	[110]
Red wine	4.95	8.89					[111]
Red wine	1.71	7.74					[112]
Red wine (var. Touriga Nacional)	1.49–1.15	7.39–15.25	50.70–67.00				[113]
Red wine (var. Trincadeira)	0.35–1.63	2.21–6.14	17.12–81.64				[113]
Red wine (var. Cabernet Sauvignon)	0.55–3.04	2.78–8.75	26.13–68.92				[113]
Red wine (var. Castelão)	0.55–3.04	2.78–8.75	26.13–68.92				[113]
Red wine (var. Syrah)	1.27–2.88	6.59–22.83	42.79–100.23				[113]
Red wine (var. Nero d’Avola)	1.96–3.02	0.70–1.23	3.23–5.14				[114]
Red wine (young)	20			75	-	-	[106]
Red wine (aged)	10			100	25	-	[106]
	White						
White wine	0.428	0.447		-	-	-	[110]
White wine	0.15	0.35	1.27				[111]
White wine	0.73	2.11	8.28				[115]
White wine (var. Grillo)	0.13–0.58	0.10–0.15	0.23–0.71				
White wine (young)	2.5			2	-	-	[106]
White wine (aged)	1.5			2.5	10	-	[106]
	Sparkling wine						[116]
White sparkling wines	0.28	2.04	4.13	6.17			[116]
Red sparkling wines	1.12	5.37	38.17	43.54			[116]

**Table 3:** Fruit juice proanthocyanidin content (fresh weight basis, mg/100 mL) [142].

	Strawberry	Saskatoon	Raspberry	Wild Blueberry
Monomer	104.68	120.97	116.44	162.69
Dimer	81.48	123.33	220.88	167.53
Oligomer	219.51	308.86	171.55	275.43
Polymer	216.93	810.19	261.68	466.64
Total	622.60	1363.34	770.55	1072.29
